# Evaluation of Hydrodynamic Chromatography Coupled with UV-Visible, Fluorescence and Inductively Coupled Plasma Mass Spectrometry Detectors for Sizing and Quantifying Colloids in Environmental Media

**DOI:** 10.1371/journal.pone.0090559

**Published:** 2014-02-28

**Authors:** Allan Philippe, Gabriele E. Schaumann

**Affiliations:** Institute for Environmental Sciences, Department of Environmental and Soil Chemistry, University Koblenz-Landau, Landau, Germany; King Abdullah University of Science and Technology, Saudi Arabia

## Abstract

In this study, we evaluated hydrodynamic chromatography (HDC) coupled with inductively coupled plasma mass spectrometry (ICP-MS) for the analysis of nanoparticles in environmental samples. Using two commercially available columns (Polymer Labs-PDSA type 1 and 2), a set of well characterised calibrants and a new external time marking method, we showed that flow rate and eluent composition have few influence on the size resolution and, therefore, can be adapted to the sample particularity. Monitoring the agglomeration of polystyrene nanoparticles over time succeeded without observable disagglomeration suggesting that even weak agglomerates can be measured using HDC. Simultaneous determination of gold colloid concentration and size using ICP-MS detection was validated for elemental concentrations in the ppb range. HDC-ICP-MS was successfully applied to samples containing a high organic and ionic background. Indeed, online combination of UV-visible, fluorescence and ICP-MS detectors allowed distinguishing between organic molecules and inorganic colloids during the analysis of Ag nanoparticles in synthetic surface waters and TiO_2_ and ZnO nanoparticles in commercial sunscreens. Taken together, our results demonstrate that HDC-ICP-MS is a flexible, sensitive and reliable method to measure the size and the concentration of inorganic colloids in complex media and suggest that there may be a promising future for the application of HDC in environmental science. Nonetheless the rigorous measurements of agglomerates and of matrices containing natural colloids still need to be studied in detail.

## Introduction

The fate of natural or engineered colloids in the environment is of great concern for estimating their ecological impacts, monitoring pollutants, and developing new pollution remediation techniques [1]–[3]. One reason why the dynamics of natural colloid formation and the final state of engineered colloids released into the environment are still unknown is the lack of suitable methods for characterising colloids in environmental samples [4]–[7]. The important parameters of the colloidal suspensions to be obtained are average size, dispersion width, elemental composition, concentration, structure (agglomerate, single-free or embedded particles), phase, charge, and coating, which require dedicated techniques and methodology [5].

The composition of environmental matrices is highly complex and variable. Inorganic ions, natural organic matter (NOM), living organisms, and inorganic particles can interact with colloids and lead to homo- or hetero-agglomeration and modify their surface properties [8]. An ideal detection and characterisation technique should therefore distinguish the targeted particles from the natural colloids, and remain accurate at realistic concentrations (ng L^−1^) [6], [9].

Microscopy is often considered to be tedious and time consuming due to the high number of pictures required for the statistical analysis [5], [10]. However, they have the advantage of giving information about elemental composition or physical properties on a single particle basis [4]. Dynamic light scattering (DLS) and multi-angle light scattering are used routinely, are non-invasive and easy to carry out [11]. These techniques are unfortunately based on advanced theories that cannot be easily and conclusively applied to complex polydisperse samples [11]. Nanoparticle tracking analysis (NTA) has better size resolution than DLS and a very low detection limit, but it has a more limited size range [12]. Analytical centrifugation requires information concerning the shape of the particles if the size distribution needs to be calculated using the mass and the density [13]. This information is unfortunately difficult to obtain in the practice. Field-flow fractionation (FFF) techniques are now widely used and have generally a high resolution [14]. However, these techniques often require long method developments and long durations [14]. Size-exclusion chromatography (SEC) is efficient and quick but its size range is limited and affinity between colloids and stationary phase can be significant due of the high packing surface area [14], [15].

Hydrodynamic chromatography (HDC) is one of the most promising methods as it provides reliable size separation that is largely independent from the matrix. The separation mechanism is based on different samplings of the flow velocity profile due to differences in the effective diameter [16]. The non-porous packing (polystyrene microspheres) limits potential interactions with the analytes rendering HDC advantageous compared to SEC [17]. Detailed comparisons of HDC with transmission electron microscopy [18] and with asymmetrical-flow FFF (AF4) [19] demonstrated that HDC is reliable for the size determination of multimodal dispersions and that it was faster and has greater recoveries than AF4, while the size resolution was much lower.

The detectors used with HDC are traditionally differential refractometers [17] or UV detectors [16], [20]. Other detection methods include particle-counting detection using laser scattering [21], multi-angle light scattering, DLS and viscosimetry [22]. Unfortunately, the detection limit of these detectors is well above ng L^−1^. Tiede *et* *al*. developed a method for the analysis of engineered nanoparticles in activated sludge using an ICP-MS-detector [23], [24]. The ICP-MS detector allows the elemental composition of the particles to be analysed at the same time as the size. For most elements its detection limit is in the ng L^−1^ range. The signal of the ICP-MS detector has been shown to be correlated with the quantity of ions produced as the particles pass through the plasma. This quantity is supposed to be independent of size since particles smaller than 500 nm are completely atomised in the plasma [25]. For this reason, it can be assumed that the number of counts registered by the detector is proportional to the number of atoms passing through the detector and thereafter to the mass of the particles.

Thus, an HDC chromatogram obtained with an ICP-MS detector can – with the help of a calibration curve - be interpreted as the mass-weighted distribution of the injected sample. If the shape and the elemental density of the particles are known, a particle number distribution can be calculated. This is unfortunately rarely the case for environmental samples. From the mass-weighted distribution, the retention time of the peak can then be interpreted as the modal distribution modus, the peak area as the elemental concentration and the peak width as the distribution width if the particle diffusion process can be omitted from the calculation [26]. This interpretation is no longer correct when the ratio between the size and the elemental mass is not known. In such cases the interpretation of the HDC-chromatogram should remain on a qualitative basis unless additional data from other detection methods are available.

Despite the promising potential of HDC-ICP-MS coupling method, some questions remain open. The effect of changes in the flow rate and the eluent composition are reported for organic colloids only, although they are important for evaluating the versatility of the method. An optimised eluent composition was already proposed by McHugh *et al*. [27]. The use of pure water as eluent leads to poor reproducibility and low recovery [27], [28]. Ionic and non-ionic surfactants increase colloidal stability, therefore preventing agglomeration, and significantly increase the recovery of colloids. A buffer is added to set the ionic strength and pH. Small [29] and later researchers [30] demonstrated that at high ionic strength the electrostatic interactions between colloids and the packing surface cannot be neglected and lead to low separation efficiency. Furthermore, ICP-MS allows for parallel measurement of size distribution and concentration. However, this combination has not yet been studied for coupling with HDC.

Colloids are expected to be present in the environment in the form of homo- or hetero-agglomerates [31]. For this reason, it is necessary to verify that agglomerates maintain their shape while passing through in the HDC column. Indeed, the surfactants could cover the surface of the particles and destabilise agglomerates [32], and shear forces provide enough energy to break down weak agglomerates [15], [33].

The objective of this study was to optimise and validate the use of HDC-ICP-MS for the characterisation and quantification of inorganic colloids in environmental samples. We coupled HDC with ICP-MS, UV-visible and fluorescence detectors (UVD and FLD respectively) in order to extend the available information on the colloidal and organic components. Commercial citrate-stabilised silver and gold nanoparticles were investigated in an artificial surface water solution containing standard moderately soft water and humic acid. We used this method to detect TiO_2_ and ZnO nanoparticles contained in commercially available sunscreens.

## Materials and Methods

### Chemicals

All Chemicals obtained from the suppliers were used without further purification. Milli-Q water (MQW, resistivity = 18 MΩ cm) was used for all dilutions and the sample preparations. The moderately soft water (MSW) solution contained: NaHCO_3_ (p.a., Merck), CaSO_4_⋅2H_2_O (p.a., Merck), MgSO_4_⋅7H_2_O (p.a., Roth), and KCl (p.a., Merck) dissolved in MQW at the following concentrations: 96, 60, 122, 86, and 4 mg L^−1^ respectively. Humic acid was provided as a solution of concentrated potassium salt solution (concentration: 18% w/w) by HuminTech under the name Humin-P 118. All ions described as standards for the ICP-MS (Au^3+^, Ag^+^, Rh^3+^, etc.) were provided by CPI International and were certified reference material.

### Reference Colloids

Standard citrate-stabilised gold nanoparticles (Aldrich, nominal diameters: 5, 30, 50, 100, 150, and 250 nm, and NIST, nominal diameter: 10 nm, analytical standards certified using TEM, AFM, and DLS) were used as size calibrants with the ICP-MS detector. Polystyrene-based fluorescent particles (nominal diameters: 22, 100, 300, 520, and 1000 nm) and non-fluorescent polystyrene particles (nominal diameter: 40 nm, certified analytical material) were provided by Thermo scientific. Citrate-stabilised silver nanoparticles (nominal diameter: 40 nm), and acid-stabilised P25 TiO_2_ (nominal diameters: 22 nm) were obtained from Aldrich and Evonik respectively.

### Sunscreens

Two sunscreens designed for application on infants were purchased in September 2012 in a commercial centre. The product names were ‘Alverde Kleine Elfe Baby-Sonnenmilch’ (S1, lotion, SPF = 30) and ‘Babylove Sonnencreme’ (S2, cream, SPF = 50). According to the ingredients lists, both products contain TiO_2_ in ‘nano’ form and S2 also contains ZnO in ‘nano’ form.

### HPLC-system

Size separation was achieved at room temperature using PL-PSDA type 1 or type 2 hydrodynamic-chromatography columns (Agilent, Germany, separation range 5–300 nm and 20–1200 nm, respectively) connected to an Agilent 1200 HPLC system (Agilent, Germany) with incorporated UV-visible and fluorescence detectors. The eluent was prepared using Milli-Q water (MQW, resistivity = 18 MΩ cm), 0.536 g L^−1^ (2 mM) Na_2_PO_4_.7H_2_O (Aldrich, purity >99%), 0.5% w/w (60 mM) formaldehyde solution (Alfa Aesar, 37% w/w H_2_O, 7–8% MeOH), 0.5 g L^−1^ (1.8 mM) sodium dodecyl sulphate (Alfa Aesar), 1 g L^−1^ (3.2 mM) Brij L23 (Alfa Aesar), 1 g L^−1^ (3.2 mM) Triton X-100 (Alfa Aesar), and 50 ng L^−1^ of CsCl (Roth, >99.9%). This formulation was adapted from Mc Gowan *et* *al*. [34]. The pH was adjusted to 7.5–8 with HNO_3_ (sub-boiled) and NaOH (Merk, Titrisol). The flow-rates were 2.1 mL min^−1^ for column type 2 and 1.7 mL min^−1^ for column type 1 (pressures of 90–95 MPa and 120–130 MPa respectively). The injection needle was adjusted to sample 30 µL (unless stated otherwise) of suspension at 1 mm from the bottom of the vial.

In order to avoid any potential interference of the internal time markers with sample components, we added Cs^+^ (50 ppt) as an external marker in the eluent. The negative peak appearing on the ^133^Cs baseline in the ICP-MS signal was used as a time marker and as an internal standard for quantification. This improvement is especially useful for environmental samples.

The retention factor is the ratio of the elution time of the particles to the elution time of the marker. From the retention factor, the effective diameter was calculated by plotting a calibration curve using the gold standards described above as calibrants and performing a linear regression of the retention factor of the peak to be analysed. Improved linearity was obtained using the square root of the size as suggested by other investigators [20]. The sizes of the calibrants used to plot the calibration curve were obtained using SEM as described elsewhere [35].

### ICP-MS System

The ICP-MS detector was a quadrupole ICP-MS XSeries 2 (Thermo) equipped with a PTFE spray chamber, thermostated with a Peltier cooler and a platinum sample cone. The parameters were optimised before each run using a tuning solution containing HDC eluent and nanoparticles standards similar in size and composition to the measured particles. Typical values for these parameters are listed in **table 1**. Where possible, several isotopes of the measured element were monitored in order to overcome potential interference. The chromatograms obtained from the ICP-MS software were analysed using the freeware program Unichrom (Unichrom, available from http://www.unichrom.com, 2013).

**Table 1:** Typical ICP-MS parameters used in this study.

Extraction	L1	L2		QP focus	D1	D2	Octopole bias	L3
−329 V	−1.23 kV	−83.1 V		7.1 V	−47.1 V	−144 V	−5 V	−191.4 V
**Forward power**	**Horizontal**	**Vertical**		**DA**	**Cool**	**Aux. gas flux**	**Nebuliser gas flow**	**Hexapole bias**
1.4 kW	105 mm	35 mm		−20.4 V	13.4°C	0.82 L min^−1^	0.66 L min^−1^	−3 V
**Sampling depth**			**Nebuliser Temperature**				**Dwell time**	
108 mm			3°C				30 ms	

### Size Calibration

The calibrants used for size determination were the citrate-stabilised gold nanoparticles (30–250 nm). The core and hydrodynamic diameters of the gold particles from 50 to 250 nm were examined using SEM and NTA respectively. Further information about these analyses is available elsewhere [35].

### Flow Rate and Eluent Effect

The citrate-stabilised gold particles (5–250 nm) were used for studying the effect of flow on the HDC-column type 1 while the polystyrene particles FluoroMax (100, 300, 1000 nm) were used with the HDC-column type 2. An ionic solution of Rh^3+^ was used as an internal time marker for these experiments. To study the effect of eluent composition, the polystyrene-based calibrants FluoroMax (nominal diameters: 22–1000 nm) and a rhodium ions standard solution were measured using the HDC-column type 2. To investigate the eluent composition effect on the hydrodynamic radius of colloids measured with DLS, we choose the non-fluorescent polystyrene (40 nm), the TiO_2_ (22 nm) and the Ag (40 nm) particles.

### Dynamic Light Scattering

A Delsa Nano C particle analyser from Beckmann-Coulter (laser wavelength: 658 nm, scattering angle: 165°, temperature: 20°C) was used for the light scattering measurements. The Z-average hydrodynamic diameter was calculated using the CONTIN method [11]. The laser position was 6.15 mm above the bottom of the vial. Polystyrene cuvettes were used. The stable suspensions were measured three times over 60 s accumulation times.

### Agglomeration Experiments

Agglomeration experiments were conducted to test the ability of the HDC to analyse relatively weak agglomerates. The effective diameter was monitored over time using HDC. We chose polystyrene colloids (non-fluorescent, 40 nm) because their agglomerates are known to be easily broken down [36], [37]. Previous experiments carried out in our laboratory confirm that the agglomerates formed by polystyrene nanoparticles broke down more easily than agglomerates of inorganic colloids like TiO_2_ or Ag (unpublished data). Suspensions were measured before adding salt and these values correspond to the time zero point. 25 µL of polystyrene suspension was diluted in 1.5 mL MQW and 30 µL of 1 M CaCl_2_ was added. After brief homogenisation (shaken three times overhead), the vials were placed in the autosampler and measurements were started. The first sample was injected 2 min 50 s after adding salt. The injection volume was 5 µL. The absorption wavelength of the UVD was 256 nm.

### Determination of the Elemental Concentrations of the Gold Calibrants

150 µL of nitric acid (65%, sub-boiled) and 310 µL of hydrochloric acid (37%) were added to 100 µL of the suspension to be measured. After one hour at room temperature, this solution was diluted in MQW to obtain a total volume of 10 mL. The concentrations of ionic gold were determined using ICP-OES (emission wavelength: 208.2 and 267.6 nm). This method was validated by measuring the certified analytical standards for ionic gold and the 10 nm gold nanoparticles (NIST).

### Humic Acid Containing Suspensions

Ag nanoparticles (40 nm, final concentration: 0.1 mg L^−1^) were added to MQW, MSW, and MSW containing 0.1–20 mg L^−1^ of humic acid. The pH levels of these solutions ranged between 6 and 7. After one hour of incubation at room temperature, samples were analysed using an HDC-ICP-MS (column type 2) without further treatment. ^107^Ag and ^109^Ag were monitored using ICP-MS detector. For the quantitative measurement of Au nanoparticles (10 nm, 10 ppb), a solution containing 5 mg L^−1^ of humic acid in MSW was used. After one hour of incubation at room temperature, the samples were analysed with ICP-MS. For these two experiments the FLD was used with the following wavelengths: λ_ext_ = 430 nm; λ_em_ = 500 nm. The absorption wavelength of the UVD was 410 nm for Ag and 517 nm for Au.

### Detection of TiO_2_ and ZnO Particles in Sunscreen

The sunscreen samples were very lipophilic and could not be suspended in water without using a surfactant or dissolving the organic matrix. In order to extract the inorganic colloids in their original state, we used a less disruptive method using Triton-X 100 as a surfactant, although a portion of the constituents remained insoluble and sedimented after incubation. 50 mg of the cream/lotion were suspended in 10 mL of MQW and 50 mg of Triton X-100 was dissolved in this solution to improve the suspension of the hydrophobic compounds. The suspensions were incubated for one hour at room temperature. 1.5 mL of the supernatant was passed through a PTFE syringe filter (1 µm cut-off) in order to remove large particles that could block the HPLC system. For this reason the following detection method is qualitative, not quantitative, and is restricted to inorganic colloids smaller than 1 µm. The filtrate was transferred to a vial for HDC analysis using the type 2 column. The absorbance wavelength of the UVD was 200 nm and the ICP-MS detector monitored the following isotopes: ^46^Ti, ^47^Ti, ^48^Ti, ^64^Zn, ^66^Zn, ^68^Zn, ^27^Al, and ^28^Si.

## Results and Discussion

### Flow Rate Effects

**Figure 1:**
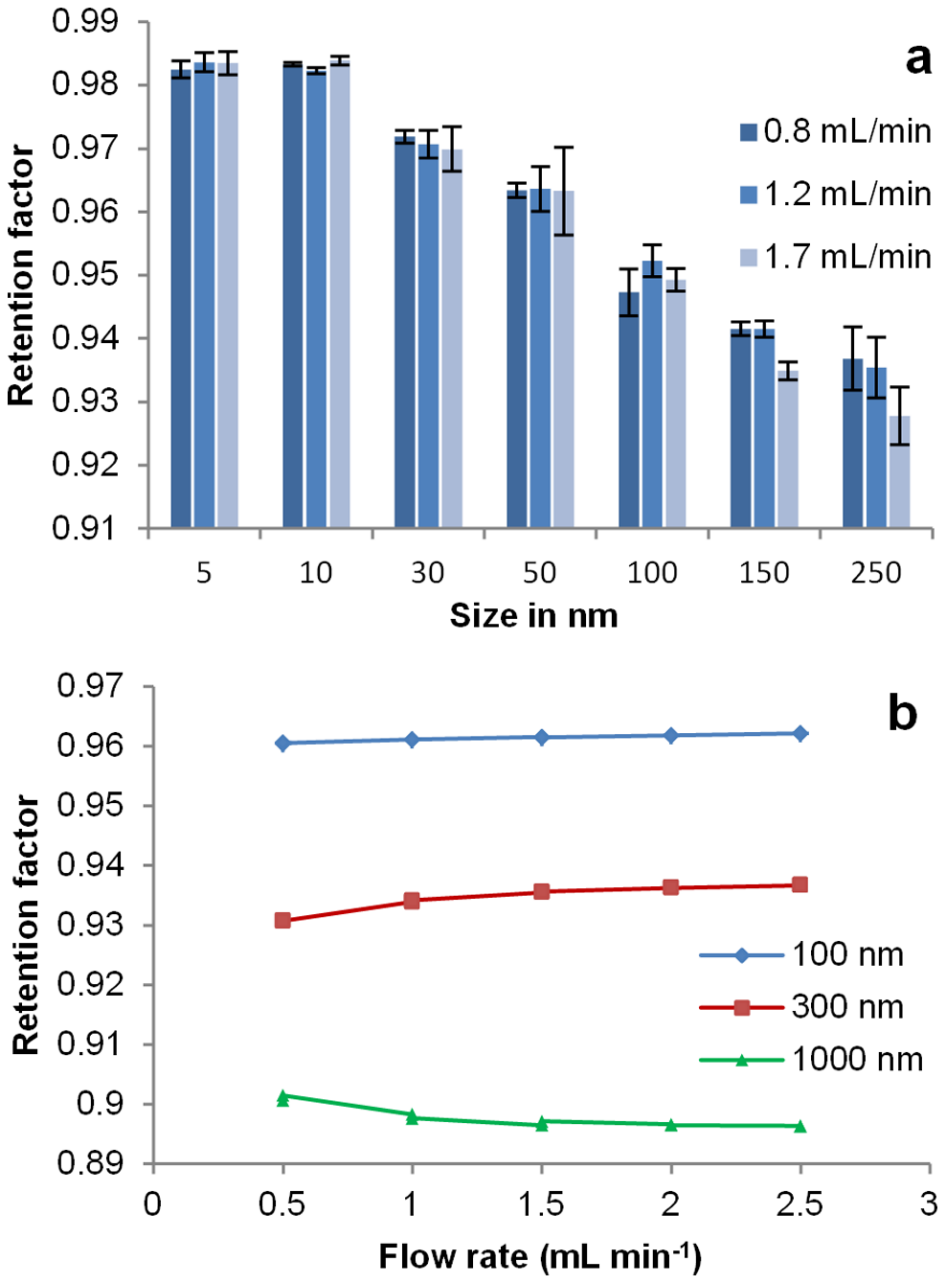
Flow rate effect. a: Retention factors of gold nanoparticle standards measured using HDC at different flow rates (column type 1; 3 replicates for 1.7 mL min^−1^ and 2 replicates for the other flow rates and); b: Retention factors of polystyrene standards (nominal diameters: 100 nm, 300 nm and 1000 nm) measured at different flow rates (column type 2, two replicates per suspension and flow rate value). The lines are provided for ease only.

Flow rate optimisation is a crucial parameter in several HDC applications (e.g. fractionation, coupling with other separation systems, etc.) and can be decisive if the analytes are sensitive to shear forces [15]. According to the basic HDC theory, flow rate should not affect separation efficiency [16]. In order to assess the validity of this assumption for heavier inorganic colloids, the retention factors of gold and polystyrene particles were measured at different flow-rates. **Figure 1a** shows the retention factors of gold nanoparticle calibrants for three different flow rates, ranging from 0.8 mL min^−1^ to 1.7 mL min^−1^. As expected, the retention factors decreased as particle size increased. The retention factors decreased as the flow rate increased for particles with a diameter equal to or greater than 150 nm, while for smaller particles the differences were in the range of the confidence intervals. Retention factors were measured in the same way for polystyrene standards at five different flow rates. **Figure 1b** shows the dependence of the retention factors on the flow rate for polystyrene colloids measuring 100 nm, 300 nm and 1000 nm respectively. For all dispersions, the retention factors varied less than 1% over the whole range of flow rates tested. The 100 nm and 300 nm dispersions were less separated at higher flow rates while the 1000 nm dispersion was better separated from the 100 nm and 300 nm dispersions.

The distribution half widths of different standard dispersions were also measured using the chromatograms and compared at different flow rates (results not shown). Even with the smallest diameter (5 nm), and consequently the highest diffusion coefficient, no significant variations were observed in the half width (0.175±0.008 mL). Therefore we consider the longitudinal diffusion of the eluted particles to be a minor contributor to the broadness of the peaks in accordance with the literature [16], [38].

These data demonstrate that elution rate is not decisive in the separation efficiency of HDC for gold or polymeric particles. Therefore, this parameter can be adapted freely according to analyst’s needs. For instance, if fast measurements are required or if interactions with the packing are expected throughout elution, high flow rate (2.1 mL min^−1^ for type 2 columns and 1.7 mL min^−1^ for type 1 columns, eight-minute measurement times) should be chosen. Lower flow rate should be chosen if shear forces need to be minimised.

### Eluent Composition Effects

**Figure 2:**
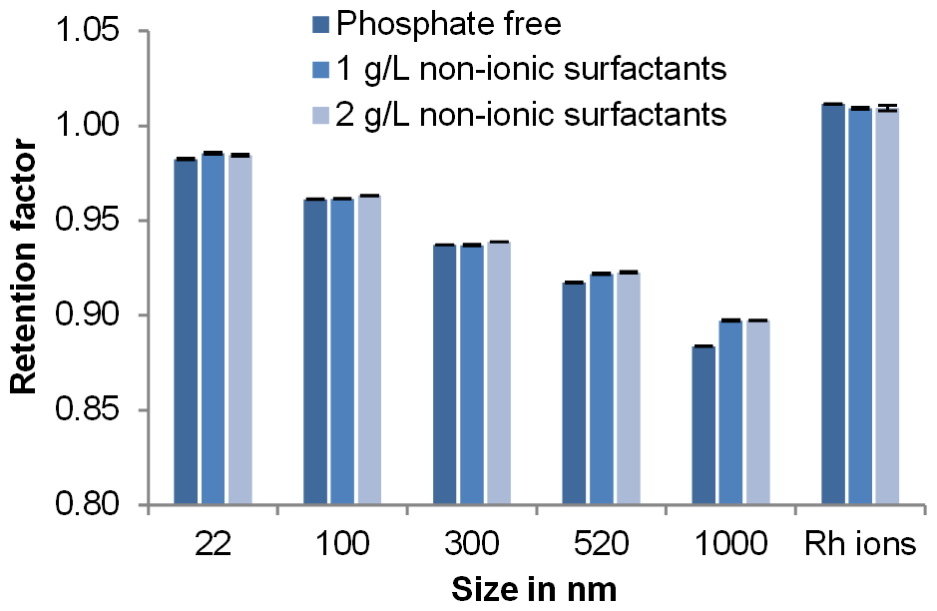
Eluent composition effect. Retention factors of polystyrene standards measured using HDC in three different eluents (column type 2, three replicates).

Complex environmental samples frequently contain substances which can interact with the eluent components. Therefore it is important to determine whether the eluent composition can be adapted without decreasing the column performance. Retention factors of polystyrene calibrants of different sizes and an Rh^3+^ solution were measured in different eluents: one with the optimised composition from McHugh *et al*. [27], one containing no phosphate, and one containing a lower concentration of surfactants (See **figure 2**). Retention factors again decreased as size increased and the ions had the highest retention factor. Retention factors for all sizes did not vary significantly when the concentration of non-ionic surfactants was doubled, suggesting that this parameter does not influence the separation efficiency. For particles larger than 300 nm, retention factors obtained in the eluent without buffer were lower than in eluents containing buffer. This indicates that the absence of phosphate in the mobile phase actually allowed slightly better separation of the particles larger than 300 nm from the ionic standard. Therefore, the mobile phase composition can be adapted to complex samples. No loss of recovery was observed with gold particles when using buffer-free eluent compared with a buffer-containing eluent, but around 20% fewer gold ions were recovered indicating a stabilisation effect of phosphate ions on gold ions throughout the elution. Composition should thus be optimised for each sample type if quantitative analyses are required.

**Figure S1** (supporting information) shows the Z-average hydrodynamic diameter determined using DLS of Ag^0^, P25 TiO_2_, and polystyrene particles suspended in water and in eluent. DLS was used because of its superior accuracy in measuring the hydrodynamic diameters of these nanoparticle suspensions compared to NTA. The differences between the sizes measured under various solution conditions were in the range of the confidence intervals. At the concentration used in the HDC eluent, the surfactants thus do not change the hydrodynamic diameter significantly and, therefore, the presence of surfactants does not bias the sizes measured using HDC.

### Measurement of Agglomerates

**Figure 3:**
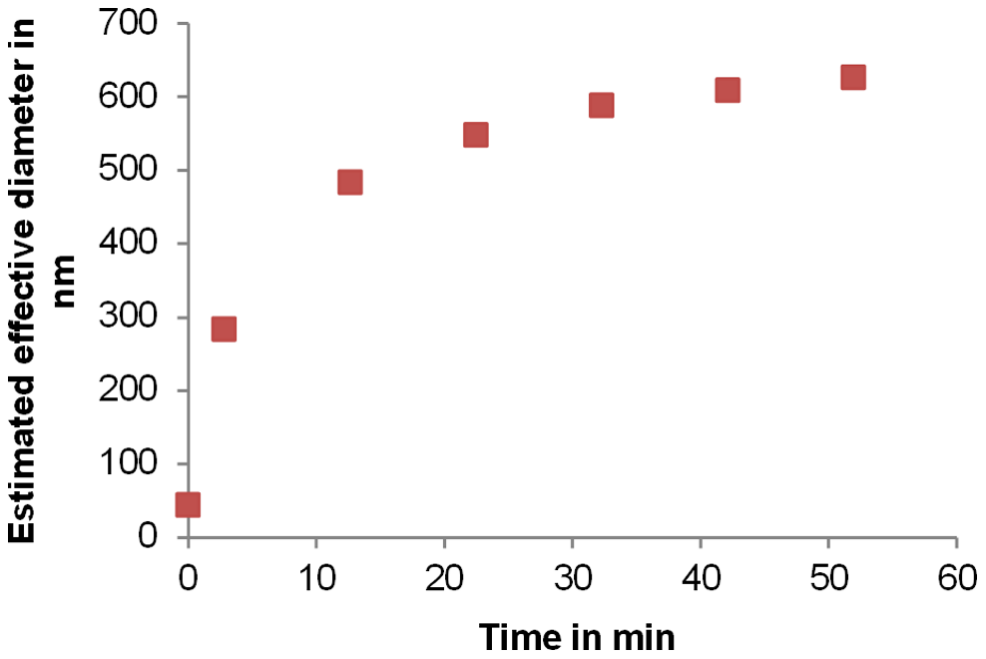
Agglomerates measurements. HDC time-resolved measurements of a latex suspension induced to agglomerate by adding CaCl_2_. Time was measured as of the addition of salt. The time zero point corresponds to the initial suspension before adding salts. The wavelength for the UV detector was 256 nm.

In order to produce agglomerates, CaCl_2_ was added to a polystyrene standard suspension. Their size was measured over the time using HDC-UVD (**figure 3**). As expected, particle size increased over time and stabilised between 600 nm and 700 nm after 1 h indicating a decrease in the agglomeration rate in accordance with results obtained for similar agglomerating systems [39]. The chromatographic peaks were symmetrical such that we can exclude disagglomeration inside the column but we cannot make any conclusions about possible breakage at the entry of the column through filtration effects or about further agglomeration that could be induced by the mixing of the particles during injection and elution [15]. Thus, the analysis of weak agglomerates by HDC is possible in accordance with Small *et al*. [29]. However, further investigation is needed to measure to what extent agglomerates breaking down or mixing effects can bias the size estimation obtained using HDC.

### Determination of the Concentration using the ICP-MS Detector

In order to verify the feasibility of quantitative measurements using an ICP-MS detector, a set of calibration curves was calculated using three gold calibration standard suspensions and one certified standard ionic gold solution. The calibration curves obtained are presented in **figure S2** (supporting information). The peak area increased linearly with the particle and ion concentration respectively for all sizes. No particle size dependence was observed. Recoveries calculated by measuring the same samples with and without an HDC-column fell between 90 and 98% for all sizes and concentrations. These values are slightly higher than those reported in the literature for similar particles [19] and allow us to have a good level of confidence in the robustness of the method for the analysis of more complicated samples. The concentration determination was validated for 10 nm standard gold particles (NIST) in a simulated surface water solution containing moderately soft water and humic acid. We used the same particles as concentration calibrants. Spiking 10 ppb of nanoparticle into this solution resulted in a recovery of 9.43±0.45 ppb. This suggests that the determination of the element concentration of colloids in environmental media using HDC-ICP-MS is reliable. However, other systems, like silver or TiO_2_ for instance, need also to be further tested for their quantitative analysis with HDC-ICP-MS.

### Analysis of Silver Nanoparticles Interaction with Humic Acid in Moderately Soft Water

**Figure 4:**
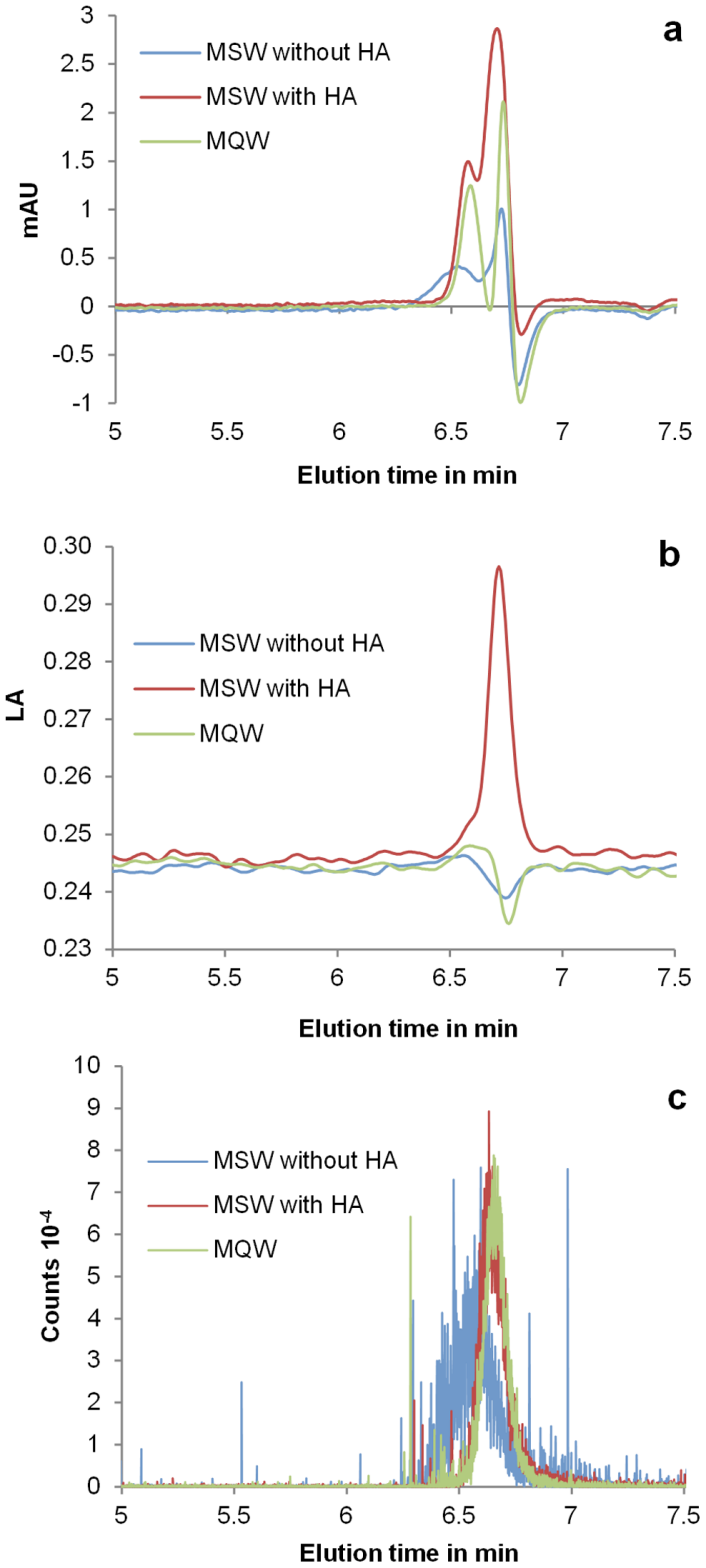
HDC-UVD-FLD-ICP-MS chromatograms of silver nanoparticles in synthetic surface water. HDC chromatograms of silver nanoparticles (core diameter: 40 nm) in MQW, MSW, and MSW with 18 mg L^−1^ of humic acid. a: ^107^Ag signal from ICP-MS detector; b: UV-VIS signal (λ_abs_ = 410 nm); c: fluorescence signal (λ_ext_ = 430 nm, λ_em_ = 500 nm). The delay between the UV-VIS detector and the ICP-MS detector was around 4 s. The dwell time of the quadrupole detector was 10 ms for all measured elements.

To test the potential of the UVD, FLD, and ICP-MS combination for investigating nanoparticles in NOM-containing samples, citrate-stabilised silver nanoparticles (nominal diameter: 40 nm) were suspended in a typical aqueous matrix containing ions and humic acid, the later at different concentrations. The UVD was optimised to detect silver nanoparticles (Plasmon resonance: 410 nm) and the FLD for detecting humic acid (λ_ext_ = 430 nm, λ_em_ = 500 nm). The ICP-MS detector was tuned to detect ^107^Ag. As examples, the chromatograms sets for a suspension containing 5 mg L^−1^ of humic acid, the initial suspension in MQW, and a suspension in MSW containing no NOM are presented in **figures 4a–c**.

Two peaks are present on the three UV-VIS chromatograms. The consideration of the retention times allows us to identify the first peak as particles and the second as ions or molecules present in the commercial solution and third to humic acid. For the solution containing humic acid, these two peaks are poorly resolved. On the chromatogram obtained using the FLD detector, only one peak can be seen when humic acid was present at a retention time corresponding to ions or molecules and to the second peak observed on the UVD chromatogram. This signal can be attributed to free humic acid since the detector wavelengths were optimised to detect humic acid specifically and silver nanoparticles do not display any fluorescence. The fluorescence peak areas of the humic substance did not vary in the presence or absence of nanoparticles. This suggests that the amount of humic acid absorbed on the particles was negligible compared to the dissolved amount. The chromatogram obtained with the ICP-MS detector showed one ^107^Ag peak at the retention time corresponding to particles and to the first peak observed on the UVD chromatogram. This peak thus corresponds to the silver nanoparticles. The observed spikes can correspond to large single silver particles, agglomerates, or multiple particles detected during the same dwell time.

**Figure 5:**
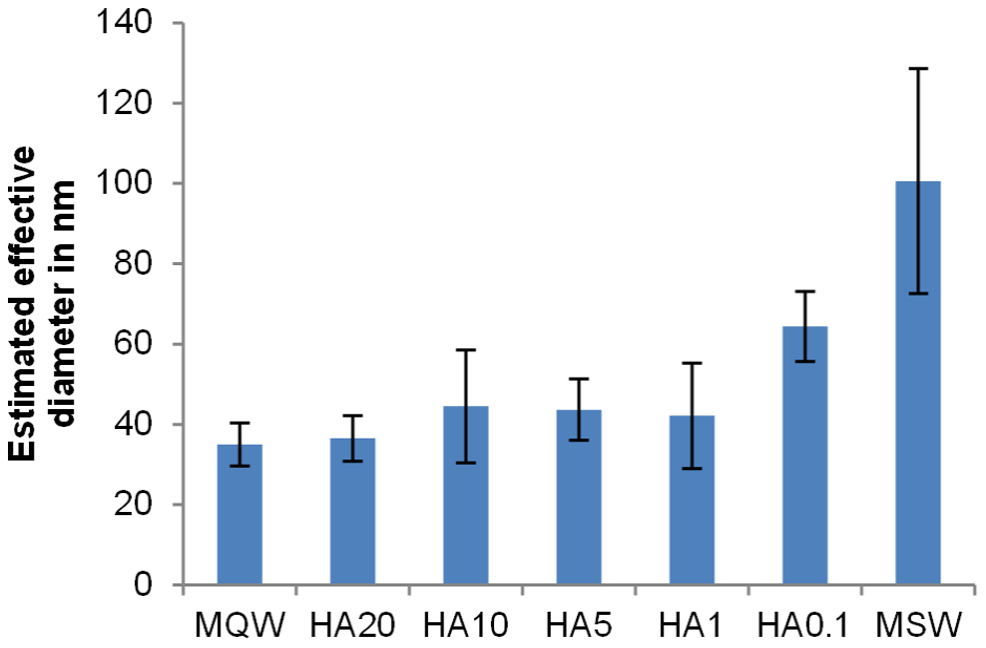
Effective diameters of silver nanoparticles in synthetic surface water. Estimated effective diameter using HDC-ICP-MS of citrate-stabilised silver nanoparticles (nominal diameter 40 nm) in different media: MQW and MSW containing X mg L^−1^ of humic acid (HAX) after three hours of incubation at room temperature. The error bars represent the confidence intervals at 95% calculated using three measurements.

The results of the size estimations of the silver nanoparticles in solutions containing humic acid at different concentrations using gold calibrants are summarised in the **figure 5**. Colloids suspended in MQW had effective diameter values comparable to the provider nominal size of 40 nm. In MQW and in solutions containing humic acid with concentrations of 1 to 20 mg L^−1^, no significant difference between the sizes of the nanoparticles was observed. However, larger sizes were measured in the solution containing 0.1 mg L^−1^ NOM and in the solution containing ions but no NOM, indicating particles agglomeration due to high ionic strength. Stabilisation of silver nanoparticles induced by humic acid was thus clearly demonstrated for humic acid concentrations above 0.1 mg L^−1^ in accordance with the literature [40]. Beneath this concentration, the amount of humic acid was thus too small to provide complete stabilisation. This demonstrates that HDC-UVD-FLD-ICP-MS is capable of analysing silver nanoparticles in solutions containing several ions and NOM.

### Detection of TiO_2_ and ZnO Colloids in Commercial Sunscreens

The same detector combination was applied for detecting TiO_2_ and ZnO engineered colloids in two sunscreens. The particles used in these formulations were unknown since no precise information was given by the suppliers, but one example of commonly used TiO_2_ nanoparticles in cosmetic products was characterised by Labille *et al* [41].

**Figure 6:**
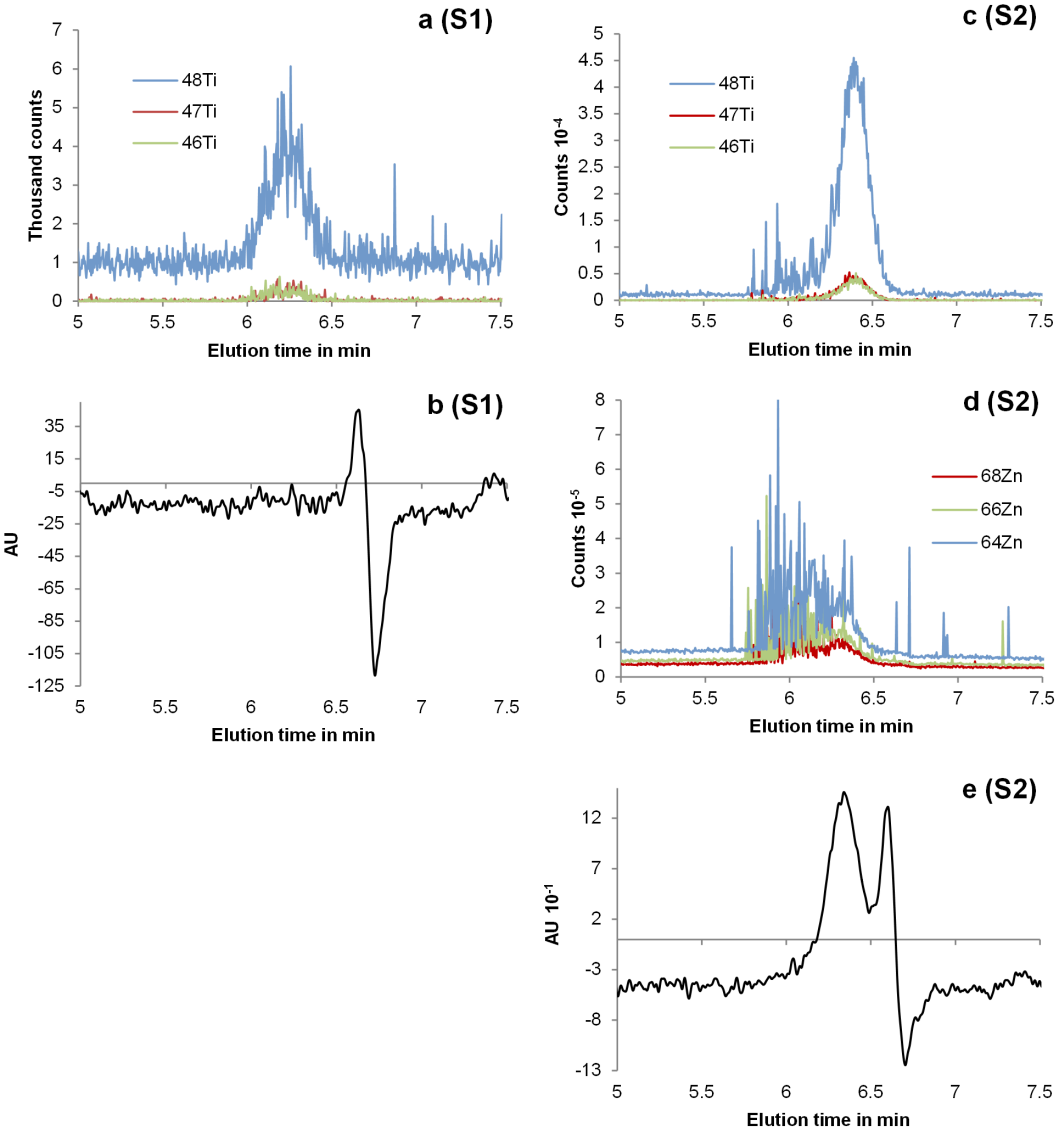
HDC-UVD-ICP-MS chromatograms of sunscreens extracts. HDC chromatograms of the colloids extracted from S1 (a and b) and S2 (c, d, and e). a and c: Titanium signal (three isotopes); b and e: UV-signal (absorption wavelength: 200 nm); d: Zinc signal (three isotopes). The time delay between the UVD and the ICP-MS detector was around 4 s.

**Figure 6a–e** shows HDC-UVD-ICP-MS chromatograms obtained for the colloids extracted from S1 and S2. Simultaneous measurements of three different Ti and Zn isotopes allowed us to avoid potential interpretation errors due to interferences in the ICP-MS detector. Titanium was detected in both samples. The particles containing Ti in S1 were revealed to have an effective diameter of 150–1000 nm with a distribution mode (most frequent size) of 500 nm and in S2 to have an effective diameter of 80–900 nm with a distribution mode of 230 nm. The UVD chromatogram (λ_abs_ = 200 nm) of S1 shows one peak corresponding to molecules or ions, while two peaks were observed for S2, one corresponding with ions or molecules and one with the TiO_2_ particles. The first peak height of the UV signal of S2 corresponds to the Ti signal and can be attributed to the TiO_2_ particles known to absorb light in this range [41]. Probably, the concentration of TiO_2_ in S1 was lower than the detection limit of the UV-detector explaining the absence of particle peak in the UV-chromatogram. This is confirmed by the observation that the Ti peaks heights of S1 are ten times smaller than the peaks heights of S2 on the ICP-MS chromatograms, indicating a lower concentration of Ti for S1 compared to S2. Colloidal Zn was detected only in S2, which matches with the supplier’s information. The particles containing Zn in S2 were broadly dispersed with effective diameters ranging from 120 nm to over 1.2 µm (resolution limit of the column). The presence of numerous spikes probably indicates large single particles or agglomerates, but multiple particles were detected during the same dwell time. No significant amounts of ^14^Si or ^27^Al were observed in these samples. The size estimations given here have to be treated with caution since the external size calibration is linked to the assumption that the particles are spherical, whereas the geometry and the exact composition of these colloids were unknown. Heterogeneous colloids containing inorganic and organic compounds cannot be unambiguously excluded in this case.

## Conclusion

In this article we have demonstrated the effectiveness of the HDC separation method combined with complementary detectors for determining the size and concentration of silver- and gold-based colloids in matrices containing salts and NOM. Flow rate and eluent composition can be adapted to the characteristics of the samples without significant loss of resolution. This makes this method highly flexible. Quantification of gold ions and colloids is possible but validation is necessary for other ions if interactions with the eluent or the packing are expected. Agglomerates can be measured using HDC but interpretation of the results requires further study of the behaviour of weak aggregates throughout elution before it can be evaluated with a high degree of confidence. The combination of ICP-MS, UV-VIS, and fluorescence detectors proved to be very useful for the analysis of Ag, TiO_2_, and ZnO colloids in mixtures containing high amounts of organic matter. To complete the evaluation of HDC for the analysis of environmental samples, further studies with solutions containing natural colloids (aluminosilicates, iron oxides, etc.) in addition to NOM have to be performed. Future works should also address HDC coupling with single particle analysis and with orthogonal separation methods [42].

## Supporting Information

Figure S1 **Effect of the eluent on the hydrodynamic diameter.** Z-average hydrodynamic diameters measured by DLS of three colloidal dispersions: Ag^0^ (dark blue), P25 TiO_2_ (blue) and polystyrene (light blue) with MQW (diluted 1∶8 and undiluted) or HDC eluent (E) as the solvent. The bars represent the confidence intervals at 95% calculated using three measurements.
(TIF)

Figure S2 **Calibration curves for measuring the concentration.** Calibration curves for the concentration estimation using HDC-ICP-MS with gold ions and gold particle standard solutions of different distribution sizes. The error bars represent the confidence intervals at 95% calculated using three measurements. Some of them are smaller than the dots.
(TIF)

## References

[1] Christian P, von der Kammer F, Baalousha M, Hofmann T (2008) Nanoparticles: structure, properties, preparation and behaviour in environmental media. Ecotoxicology 17: 326–343. 18459043 10.1007/s10646-008-0213-1

[2] Lead JR, Wilkinson KJ (2006) Aquatic colloids and nanoparticles: current knowledge and future trends. Environ Chem 3: 159–171.

[3] Wigginton NS, Haus KL, Hochella Jr MF (2007) Aquatic environmental nanoparticles. J Environ Monitor 9: 1306–1316. 10.1039/b712709j 18049768

[4] Burleson DJ, Driessen MD, Penn RL (2005) On the characterization of environmental nanoparticles. J Environ Sci Heal, Part A 39: 2707–2753. 10.1081/ese-200027029 15509018

[5] Hassellöv M, Readman JW, Ranville JF, Tiede K (2008) Nanoparticle analysis and characterization methodologies in environmental risk assessment of engineered nanoparticles. Ecotoxicology 17: 344–361. 18483764 10.1007/s10646-008-0225-x

[6] Simonet BM, Valcárcel M (2009) Monitoring nanoparticles in the environment. Anal Bioanal Chem 393: 17–21. 18974979 10.1007/s00216-008-2484-z

[7] Tiede K, Boxall A, Tear S, Lewis J, David H, et al (2008) Detection and characterization of engineered nanoparticles in food and the environment-a review. Food Addit Contam 25: 795–821. 10.1080/02652030802007553 18569000

[8] Aiken GR, Hsu-Kim H, Ryan JN (2011) Influence of dissolved organic matter on the environmental fate of metals, nanoparticles, and colloids. Environ Sci Technol 45: 3196–3201. 21405118 10.1021/es103992s

[9] Nowack B, Ranville JF, Diamond S, Gallego-Urrea JA, Metcalfe C, et al (2012) Potential scenarios for nanomaterial release and subsequent alteration in the environment. Environ Toxicol Chem 31: 50–59. 22038832 10.1002/etc.726

[10] von der Kammer F, Ferguson PL, Holden PA, Masion A, Rogers KR, et al (2012) Analysis of engineered nanomaterials in complex matrices (environment and biota): general considerations and conceptual case studies. Environ Toxicol Chem 31: 32–49. 22021021 10.1002/etc.723

[11] Finsy R (1994) Particle sizing by quasi-elastic light scattering. Adv Colloid Interfac 52: 79–143.

[12] Filipe V, Hawe A, Jiskoot W (2010) Critical evaluation of Nanoparticle Tracking Analysis (NTA) by NanoSight for the measurement of nanoparticles and protein aggregates. Pharm Res 27: 796–810. 20204471 10.1007/s11095-010-0073-2 PMC2852530

[13] Wohlleben W (2012) Validity range of centrifuges for the regulation of nanomaterials: from classification to as-tested coronas. J of Nanopart Res 14: 1–18. 23239934 10.1007/s11051-012-1300-z PMC3517805

[14] Fedotov PS, Vanifatova NG, Shkinev VM, Spivakov BY (2011) Fractionation and characterization of nano-and microparticles in liquid media. Anal Bioanal Chem 400: 1787–1804. 21318253 10.1007/s00216-011-4704-1

[15] Brewer AK, Striegel AM (2011) Characterizing string-of-pearls colloidal silica by multidetector hydrodynamic chromatography and comparison to multidetector size-exclusion chromatography, off-line multiangle static light scattering, and transmission electron microscopy. Anal Chem 83: 3068–3075. 21428298 10.1021/ac103314c

[16] Striegel AM, Brewer AK (2012) Hydrodynamic Chromatography. Annu Rev Anal Chem 5: 15–34. 10.1146/annurev-anchem-062011-143107 22708902

[17] Penlidis A, Hamielec A, MacGregor J (1983) Hydrodynamic and Size Exclusion Chromatography of Particle Suspensions-An Update. J Liq Chromatogr 6: 179–217.

[18] DosRamos JG, Silebi CA (1990) The determination of particle size distribution of submicrometer particles by capillary hydrodynamic fractionation (CHDF). J Colloid Interface Sci 135: 165–177.

[19] Gray EP, Bruton TA, Higgins CP, Halden RU, Westerhoff P, et al (2012) Analysis of gold nanoparticle mixtures: a comparison of hydrodynamic chromatography (HDC) and asymmetrical flow field-flow fractionation (AF4) coupled to ICP-MS. J of Anal At Spectrom 27: 1532–1539.

[20] Williams A, Varela E, Meehan E, Tribe K (2002) Characterisation of nanoparticulate systems by hydrodynamic chromatography. Int J Pharm 242: 295–299. 12176267 10.1016/s0378-5173(02)00191-6

[21] Zarrin F, Dovichi NJ (1985) Particle counting by laser light scatter for capillary hydrodynamic chromatography. Anal Chem 57: 1826–1829. 4037343 10.1021/ac00286a010

[22] Brewer AK, Striegel AM (2009) Particle size characterization by quadruple-detector hydrodynamic chromatography. Anal Bioanal Chem 393: 295–302. 18762925 10.1007/s00216-008-2319-y

[23] Tiede K, Boxall AB, Wang X, Gore D, Tiede D, et al (2010) Application of hydrodynamic chromatography-ICP-MS to investigate the fate of silver nanoparticles in activated sludge. J of Anal At Spectrom 25: 1149–1154.

[24] Tiede K, Boxall AB, Tiede D, Tear SP, David H, et al (2009) A robust size-characterisation methodology for studying nanoparticle behaviour in “real” environmental samples, using hydrodynamic chromatography coupled to ICP-MS. J of Anal At Spectrom 24: 964–972.

[25] Dubascoux S, Le Hecho I, Hassellöv M, Von Der Kammer F, Gautier MP, et al (2010) Field-flow fractionation and inductively coupled plasma mass spectrometer coupling: History, development and applications. J of Anal At Spectrom 25: 613–623.

[26] Venema E, Kraak J, Poppe H, Tijssen R (1996) Packed-column hydrodynamic chromatography using 1-micrometer non-porous silica particles. J Chromatogr A 740: 159–167.

[27] McHugh AJ, Brenner H (1984) Particle size measurement using chromatography. Crit Rev Anal Chem 15: 63–117.

[28] Small H (1974) Hydrodynamic chromatography a technique for size analysis of colloidal particles. J Colloid Interface Sci 48: 147–161.

[29] Small H, Saunders FL, Solc J (1976) Hydrodynamic chromatography a new approach to particle size analysis. Adv Colloid Interfac 6: 237–266.

[30] Prieve DC, Hoysan PM (1978) Role of colloidal forces in hydrodynamic chromatography. J Colloid Interface Sci 64: 201–213.

[31] Hotze EM, Phenrat T, Lowry GV (2010) Nanoparticle aggregation: Challenges to understanding transport and reactivity in the environment. J of Environ Qual 39: 1909–1924. 21284288 10.2134/jeq2009.0462

[32] Baalousha M (2009) Aggregation and disaggregation of iron oxide nanoparticles: Influence of particle concentration, pH and natural organic matter. Sci Total Environ 407: 2093–2101. 19059631 10.1016/j.scitotenv.2008.11.022

[33] Jarvis P, Jefferson B, Parsons SA (2005) Breakage, regrowth, and fractal nature of natural organic matter flocs. Environ Sci Technol 39: 2307–2314. 15871269 10.1021/es048854x

[34] McGowan GR, Langhorst MA (1982) Development and application of an integrated, high-speed, computerized hydrodynamic chromatograph. J Colloid Interface Sci 89: 94–106.

[35] Rakcheev D, Philippe A, Schaumann GE (2013) Hydrodynamic Chromatography Coupled with Single Particle-Inductively Coupled Plasma Mass Spectrometry for Investigating Nanoparticles Agglomerates. Anal Chem 85: 10643–10647. 24156639 10.1021/ac4019395

[36] Ouali L, Pefferkorn E (1994) Fragmentation of colloidal aggregates induced by polymer adsorption. J Colloid Interface Sci 168: 315–322.

[37] Walker HW, Bob MM (2001) Stability of particle flocs upon addition of natural organic matter under quiescent conditions. Water Res 35: 875–882. 11235882 10.1016/s0043-1354(00)00333-x

[38] Takeuchi T, Aspanut Z, Lim WL (2009) Hydrodynamic Chromatography of Silica Colloids on Small Spherical Nonporous Silica Particles. Anal Sci 25: 301–306. 19212069 10.2116/analsci.25.301

[39] Hanus LH, Hartzler RU, Wagner NJ (2001) Electrolyte-induced aggregation of acrylic latex. 1. Dilute particle concentrations. Langmuir 17: 3136–3147.

[40] Chinnapongse SL, MacCuspie RI, Hackley VA (2011) Persistence of singly dispersed silver nanoparticles in natural freshwaters, synthetic seawater, and simulated estuarine waters. Sci Total Environ 409: 2443–2450. 21481439 10.1016/j.scitotenv.2011.03.020

[41] Labille J, Feng J, Botta C, Borschneck D, Sammut M, et al (2010) Aging of TiO2 nanocomposites used in sunscreen. Dispersion and fate of the degradation products in aqueous environment. Environ Pollut 158: 3482–3489. 20346555 10.1016/j.envpol.2010.02.012

[42] Lespes G, Gigault J (2011) Hyphenated analytical techniques for multidimensional characterisation of submicron particles: A review. Anal Chim Acta 692: 26–41. 21501709 10.1016/j.aca.2011.02.052

