# Supplementary figures and images for: Evaluation of Hydrodynamic Chromatography Coupled with UV-Visible, Fluorescence and Inductively Coupled Plasma Mass Spectrometry Detectors for Sizing and Quantifying Colloids in Environmental Media
Source: PLoS One. 2014 Feb 28;9(2):e90559. doi: 10.1371/journal.pone.0090559 (PMC3938767; doi:10.1371/journal.pone.0090559)

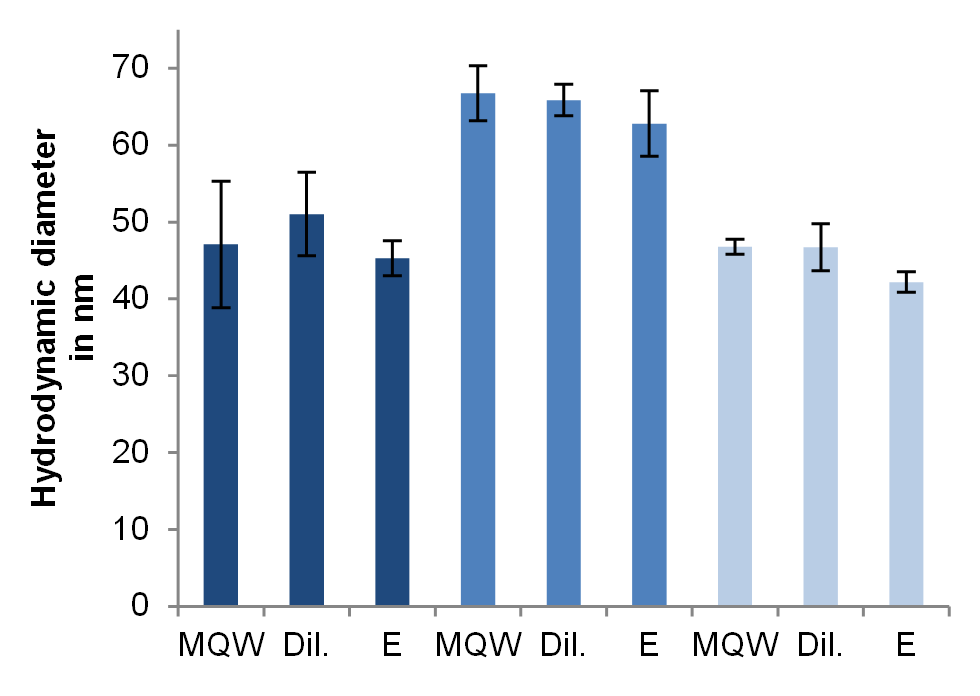

Supplement: Figure S1 — Effect of the eluent on the hydrodynamic diameter. Z-average hydrodynamic diameters measured by DLS of three colloidal dispersions: Ag0 (dark blue), P25 TiO2 (blue) and polystyrene (light blue) with MQW (diluted 1∶8 and undiluted) or HDC eluent (E) as the solvent. The bars represent the confidence intervals at 95% calculated using three measurements. (TIF) [file pone.0090559.s001.tif]

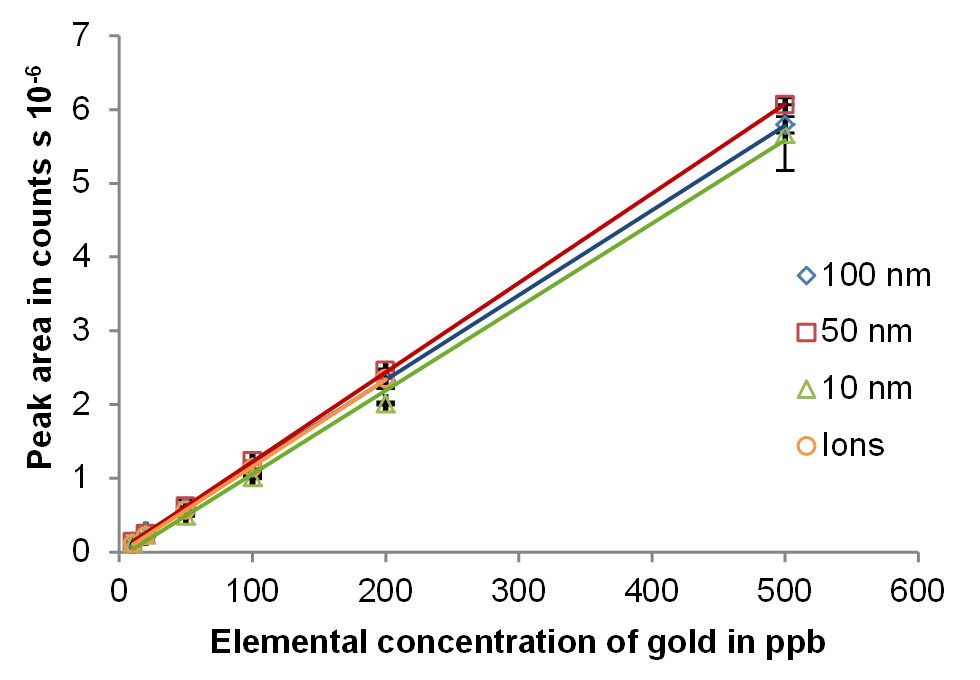

Supplement: Figure S2 — Calibration curves for measuring the concentration. Calibration curves for the concentration estimation using HDC-ICP-MS with gold ions and gold particle standard solutions of different distribution sizes. The error bars represent the confidence intervals at 95% calculated using three measurements. Some of them are smaller than the dots. (TIF) [file pone.0090559.s002.tif]
